# Impact of third-generation cephalosporin and carbapenem resistance profiles on the mortality of patients with *Enterobacter cloacae* complex bloodstream infections: a retrospective multicentre cohort study

**DOI:** 10.1093/jacamr/dlag172

**Published:** 2026-08-25

**Authors:** Jaysa Pizzi, Beatriz Arns, Emerson D S Hoffmann, Rodrigo C da Fonseca, Greici T Gunzel, Julia S de Villa, Bárbara D P Modesti, Guilherme G L Sório, Helen D S Feiten, Alexandre P Zavascki

**Affiliations:** Programa de Pós-graduação em Ciências Médicas, Universidade Federal do Rio Grande do Sul, Porto Alegre 90035-004, Brazil; Infectious Diseases Service, Hospital de Clínicas de Porto Alegre, Porto Alegre, Brazil; Programa de Pós-graduação em Ciências Médicas, Universidade Federal do Rio Grande do Sul, Porto Alegre 90035-004, Brazil; Infectious Diseases Service, Hospital de Clínicas de Porto Alegre, Porto Alegre, Brazil; Infectious Diseases and Infection Control Service, Hospital Moinhos de Vento, Porto Alegre, Brazil; Infectious Diseases and Infection Control Service, Hospital Moinhos de Vento, Porto Alegre, Brazil; Infectious Diseases and Infection Control Service, Hospital Moinhos de Vento, Porto Alegre, Brazil; Infectious Diseases Service and Infection Control Service, Hospital Nossa Senhora da Conceição, Porto Alegre, Brazil; Infectious Diseases Service and Infection Control Service, Hospital Nossa Senhora da Conceição, Porto Alegre, Brazil; Infectious Diseases Service and Infection Control Service, Hospital Nossa Senhora da Conceição, Porto Alegre, Brazil; Infectious Diseases and Infection Control Service, Hospital Moinhos de Vento, Porto Alegre, Brazil; Infectious Diseases Service and Infection Control Service, Hospital Nossa Senhora da Conceição, Porto Alegre, Brazil; Clinical Pharmacy Department, Hospital Moinhos de Vento, Porto Alegre, Brazil; Infectious Diseases Service, Hospital de Clínicas de Porto Alegre, Porto Alegre, Brazil; Infectious Diseases Service and Infection Control Service, Hospital Nossa Senhora da Conceição, Porto Alegre, Brazil; Department of Internal Medicine, Universidade Federal do Rio Grande do Sul, Porto Alegre, Brazil

## Abstract

**Objectives:**

To evaluate 30-day mortality in *Enterobacter cloacae* complex (Ecc) bloodstream infections (BSIs) according to third-generation cephalosporin and carbapenem resistance profiles, and to investigate factors contributing to the association between antimicrobial resistance and this outcome.

**Methods:**

Multicentre retrospective cohort including adults with healthcare-associated Ecc BSI from four Brazilian tertiary hospitals. Isolates were categorized as: third-generation cephalosporin-susceptible (3GCS), third-generation cephalosporin-resistant (3GCR), or carbapenem-resistant (CR). A hierarchical Cox model assessed the effect of resistance profiles on 30-day mortality, adjusting sequentially for: (i) baseline status, (ii) BSI-related variables and (iii) antimicrobial therapy.

**Results:**

Of 429 patients, 300 (69.9%) had 3GCS, 103 (24.0%) 3GCR and 26 (6.1%) CR Ecc BSIs. Thirty-day mortality was 22.6% (20.6%, 25.2% and 34.6%, respectively; *P* = 0.20). In unadjusted analysis, 3GCR (HR 1.10; 95% CI 0.70–1.73) and CR (HR 1.42; 95% CI 0.70–2.86) were not significantly associated with mortality. Hierarchical adjustment revealed that the association between CR and mortality was attenuated after adjustment for baseline severity, while adjustment for appropriate therapy attenuated the associations observed for both resistance profiles. Higher Charlson and Pitt scores, lower baseline glomerular filtration rate and polymicrobial BSI were independently associated with higher mortality; adequate therapy at any time was strongly protective.

**Conclusions:**

Non-statistically significant mortality rates were observed in patients with 3GCR and CR Ecc BSIs. Baseline severity and, most importantly, less frequent appropriate antimicrobial therapy attenuated the effect of resistance profiles on the outcome. Improving antimicrobial therapy against resistant Ecc isolates has great potential to reduce mortality.

## Introduction

Resistance to third-generation cephalosporin and to carbapenem in *Enterobacterales* were classified by the WHO as critical priority antimicrobial resistance profile on the first and updated bacterial priority pathogens list.^1,2^ Although research within *Enterobacterales* has predominantly focused on *Klebsiella pneumoniae*, *Enterobacter* spp. are also major causes of healthcare-associated bloodstream infections (BSIs) and were responsible for >300 000 deaths globally in 2019.^3,4^

Among *Enterobacter* species, the *Enterobacter cloacae* complex (Ecc) accounts for most clinical infections and comprises several closely related species with dynamic taxonomy, with *E. hormaechei* and *E. cloacae* being the most frequently identified among clinical isolates.^5–7^ Ecc species harbour a chromosomally encoded *ampC* gene with potential for de-repression on β-lactam exposure and frequently acquire additional β-lactamase genes, including those encoding ESBLs, via mobile genetic elements.^8,9^ As reflected in the WHO classification, third-generation cephalosporin resistance serves as a surrogate marker for the presence of ESBL and/or de-repressed AmpC β-lactamases.^1^ The production of either class A or B carbapenemases has been the main mechanism of carbapenem resistance in *Enterobacterales*,^10–12^ including Ecc isolates.^13–15^ AmpC or ESBL overexpression combined with porin loss and increased efflux activity may also be responsible for carbapenem resistance in Ecc.^13^

Most studies investigating the impact of third-generation cephalosporin and particularly carbapenem resistance among *Enterobacterales* have been majorly or exclusively composed by *K. pneumoniae*.^16–20^ Despite the paucity of clinical studies with Ecc infections, this species has been reported as a major healthcare-associated pathogen.^21–23^

The aim of this study was to evaluate 30-day mortality in healthcare-associated Ecc BSIs according to third-generation cephalosporin and carbapenem resistance profiles, and to assess the confounding effect of specific groups of variables, i.e. the association of these other variables with the resistance profiles and mortality, using a hierarchical modelling approach.

## Methods

### Study design and participants

This was a multicentric retrospective cohort study performed in four tertiary-care hospitals in Brazil from January 2012 to December 2023. Patients were included if they were ≥18 years-old and had a healthcare-associated Ecc BSI. A healthcare-associated BSI was defined by the presence of at least one blood culture collected after >2 days of hospitalization. Patients were excluded if medical recordings were unavailable. If the patient presented more than one Ecc BSI episode, only the first was included.

Screening of potentially eligible patients was conducted through queries of databases and microbiology laboratory data. Data were retrieved from electronic medical records. The study protocol was approved by the institutional review boards of all participating hospitals (CAAE 76773024.6.1001.5330, approved on 5 February 2024), and the requirement for informed consent was waived.

### Microbiology

Bacterial identification was performed using the Vitek2^®^ automatized system or MALDI-TOF (bioMérieux, France) system depending on the hospital and period of the study. Susceptibility to meropenem, ceftazidime and ceftriaxone was evaluated by disc diffusion and interpreted according to CLSI^24^ breakpoints until December 2018 and from January 2019 onwards, according to EUCAST criteria,^25^ when Brazilian laboratories adopted the EUCAST standards.^26^ Other antimicrobials were also evaluated by disc diffusion. except for polymyxin B (broth microdilution) and tigecycline (broth microdilution or gradient strip). Isolated with minimal inhibitory concentrations ≤2 and 0.5 mg/L were considered susceptible to polymyxin B and tigecycline, respectively.

Carbapenemase identification was performed by phenotypic (disc diffusion with inhibitors,^27^ NG-Test^®^ CARBA-5^28^ or improved Blue-Carba test^29,30^) or genotypic (polymerase chain reaction) tests,^31^ depending on hospital and period of the study.

### Variables and definitions

Three groups of Ecc were divided according to the antimicrobial susceptibility profile of Ecc isolates: third-generation cephalosporin and carbapenem-susceptible (for simplicity, just 3GCS); third-generation cephalosporin-resistant and carbapenem-susceptible (3GCR); and cephalosporin-resistant and carbapenem-resistant (CR). Isolates classified as intermediate or susceptible, increased exposure were considered susceptible. Third-generation cephalosporin resistance was defined as resistance to ceftazidime and/or ceftriaxone; and carbapenem resistance was defined as resistance to meropenem.^24,25^

The outcome was 30-day mortality, defined as death for any cause within the first 30 days after the BSI onset, which was considered the day of blood culture collection that yielded the growth of Ecc isolate.

Covariables addressed were classified in three categories. Variables associated with patients’ baseline status were: sex at birth, age, hospital of admission, time to BSI, hospitalization in ICU at BSI onset, Charlson comorbidity score^32^ and glomerular filtration rate (estimated by CKD-EPI equation).^33^ Variables associated with severity of BSI were: primary site of infection, polymicrobial infection, septic shock and use of mechanical ventilation at the time of BSI onset. Variables associated with antimicrobial therapy were: adequate empirical therapy, adequate therapy at any time and use of combination therapy.

The primary site of infection was defined according to the CDC/NHSN.^34^ Time to BSI was defined as the length of hospital stay before BSI onset. Polymicrobial infection was considered if a microorganism other than an Ecc isolate was also identified in the same blood cultures set that Ecc was recovered (for coagulase-negative *Staphylococcus* spp., BSI was considered only with two positive blood culture sets). The primary site of BSI was categorized based on the distribution of observed mortality rates across infection sites.

Adequate antimicrobial therapy was considered if the patient received at least one antimicrobial to which the Ecc isolate demonstrated *in vitro* susceptibility, and it was initiated within 7 days of BSI and maintained for ≥2 days. Antimicrobial therapy initiated >7 days of BSI was considered inadequate. Empirical therapy was defined as the antimicrobial regimen administered from the BSI onset (day 1) to day 3. Combination therapy was defined as the use of concomitant two or more antimicrobial agents to which the Ecc isolate demonstrated *in vitro* susceptibility for ≥2 days.

### Statistical analysis

Statistical analysis was performed using PASW Statistics for Windows v.18.0 (SPSS Inc., Chicago, IL, USA). Two separate univariate analyses were performed: one comparing variables across susceptibility profile groups (3GCS, 3GCR and CR) and another comparing variables according to 30-day mortality status (survivors versus non-survivors). The *P* were calculated using the *χ*2 test for categorical variables, and ANOVA or the Kruskal–Wallis test for continuous variables in the first univariate analysis. In the second univariate analysis, comparisons were performed using the *χ*^2^ or Fisher’s exact test for categorical variables and the Mann–Whitney exact test or Student’s *t* test for continuous variables. All tests were two-tailed, and a *P* < 0.05 was considered statistically significant.

An unadjusted hazard ratio (HR) was calculated to assess the effect of the Ecc susceptibility profile on mortality, followed by a Cox proportional hazard, which was constructed using a hierarchized framework of the variables, as proposed by Victora *et al*.,^35^and previously used by Zavascki *et al*.^36^ Variables with *P* ≤ 0.20 in any of the univariate analyses were included in the model following sequence: Step 1: variables related to the patients baseline status; Step 2: variables that remained from step 1 plus those related to BSI and Step 3 (final model): variables that remained from Steps 1 and 2 plus those related to the treatment. We used the variations of *β* coefficients of the variables under study (i.e. 3GCR and CR) as an auxiliary tool to interpret the effect of other variables at each step of analysis, which may capture effects beyond the *P.*^37,38^ The change (%) in *β* coefficient the first step was calculated according to the formula: proportional change in *β* coefficient = unadjusted *β* coefficient − step 1 adjusted *β* coefficient × 100; and the following step *n* adjusted *β* coefficient − step *n* − 1 adjusted *β* coefficient × 100. A *P* of 0.05 was defined as statistically significant and determined whether the variable would remain in the model. If the variables selected for inclusion in the model were collinear, only the variable with the lowest *P* was retained.

The rationale for the hierarchized approach was to assess which factors were potential confounding factors of the association of 3GCR and CR with mortality compared to the 3GCS group. Each step illustrates the impact of these resistance profiles on mortality after adjusting for the confounding variables included at each step, as well as for those retained from previous steps.^35^

## Results

### Patient characteristics

**Figure 1.**
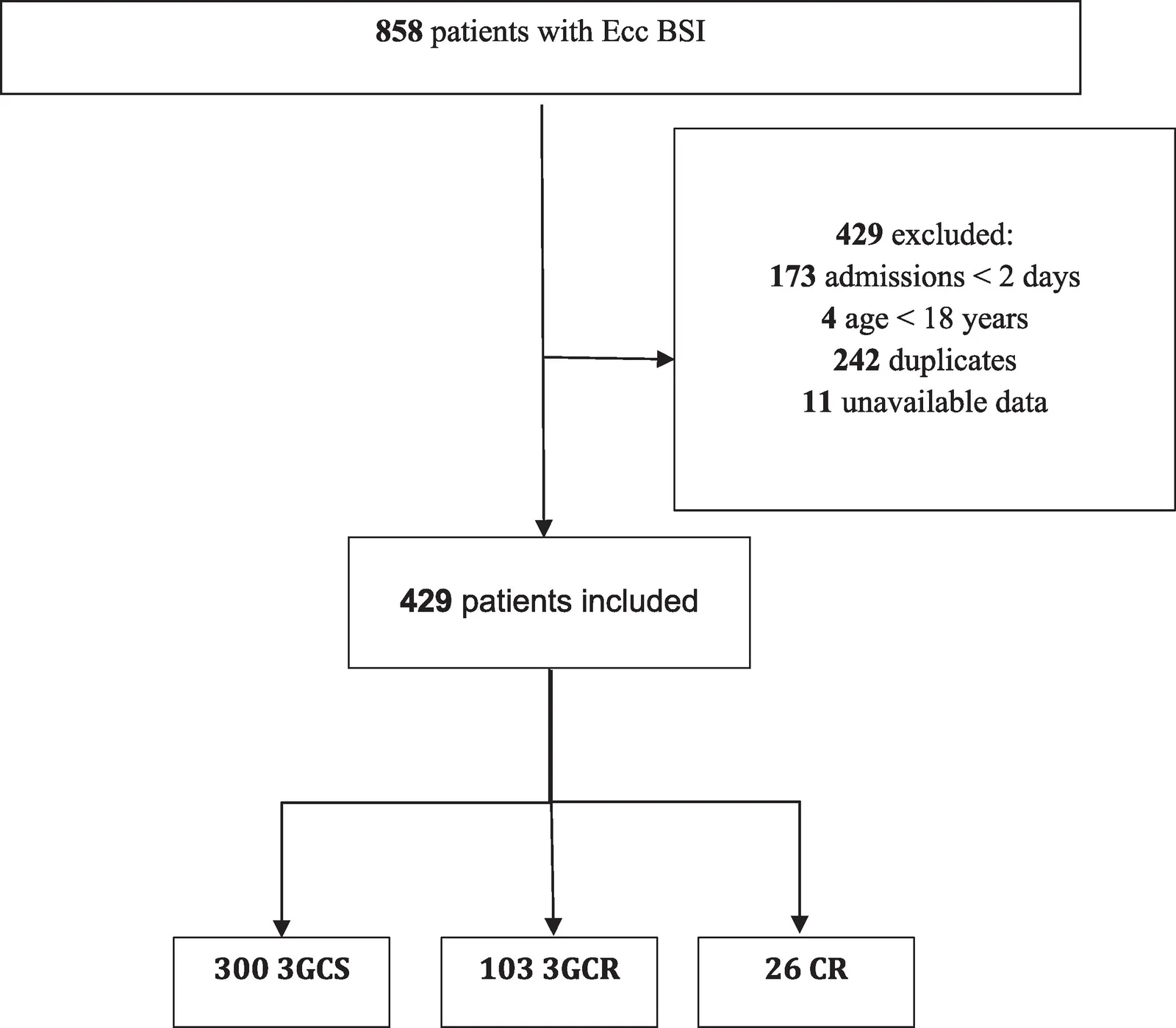
Study population flow diagram. Flowchart of patient selection for inclusion in the study. Of 858 patients with Ecc BSI, 429 were excluded, leaving 429 patients categorized in three groups according to susceptible profiles: 3GCS, *n* = 300; 3GCR, *n* = 103 and CR, *n* = 26.

A total of 858 blood cultures positive for Ecc were screened, and 429 were excluded. Of these, 242 due to duplicate isolates, 173 were from patients with a hospital stay shorter than 2 days, four were from patients under 18 years and 11 had missing data, resulting in a total of 429 patients (197 patients from Hospital B, 105 from Hospital C, 108 from Hospital D and 19 from Hospital A) included in the study (Figure 1).

The susceptibility profile of Ecc isolates was 300 (69.9%) 3GCS, 103 (24.0%) 3GCR and 26 (6.1%) CR. The characteristics of the entire cohort of patients and according to antimicrobial susceptibility profile groups are shown in Table 1. Patients of the CR group presented significantly longer lengths of hospital stay compared with those of other groups, and had non-statistically significant higher rates of Ecc BSIs onset during an ICU stay (Table 1). Patients with CR Ecc had a significantly higher proportion of high-risk primary (defined according to specific mortality by primary site, see the classification next) as a primary site of BSIs (Table 1). The frequency of appropriate empirical and at any time therapy were both significantly lower in the CR Ecc group, which also presented a significantly higher proportion of combination adequate therapy at any time. The remaining variables were generally similar among groups, with all presenting *P* values >0.20.

**Table 1.** Characteristics of patients with Ecc BSIs according to susceptibility profiles

Variable	Total (*n* = 429)	3GCS (*n* = 300)	3GCR (*n* = 103)	CR (*n* = 26)	*P*
Variables associated with patients’ baseline status					
Age (years), mean ± SD	60.7 ± 18	60 ± 18	62 ± 17.8	64 ± 16.7	0.41
Sex					0.25
Male, *n* (%)	217 (50.6)	144 (48)	59 (57.3)	14 (53.8)	
Hospital of admission					0.36
Hospital A, *n* (%)	19 (4.4)	11 (3.7)	8 (7.8)	0 (0)	
Hospital B, *n* (%)	197 (45.9)	135 (45)	48 (46.6)	14 (53.8)	
Hospital C, *n* (%)	105 (24.5)	75 (25)	22 (21.4)	8 (30.8)	
Hospital D, *n* (%)	108 (25.2)	79 (26.3)	25 (24.3)	4 (15.4)	
Charlson comorbidity index, median (IQR)	2 (1 -4)	2 (2–4)	2 (1–5)	3 (1–4.25)	0.80
Baseline CLCr (mL/min), median (IQR)	66 (42–104)	79.2 (37.3–101.9)	65.7 (31.8–101.8)	51.7 (24.3–98.8)	0.34
BSI onset at ICU, *n* (%)	100 (23.3)	68 (22.7)	21 (20.4)	11 (42.3)	0.06
Variables associated with BSI					
Pitt bacteraemia score, median (IQR)	0 (0–2)	0 (0–2)	0 (0–1)	1 (0–5)	0.22
Time to BSI (days), median (IQR)^a^	14 (7–25)	13 (7–24)	15 (8–27)	22 (12.5–41.7)	0.05
Primary site infection					0.003
Abdominal, *n* (%)	91 (21.2)	72 (24)	15 (14.6)	4 (15.4)	
Urinary, *n* (%)	33 (7.7)	17 (5.7)	16 (15.5)	0 (0)	
Catheter-associated, *n* (%)	92 (21.4)	66 (22)	21 (20.4)	5 (19.2)	
Pulmonary, *n* (%)	76 (17.7)	46 (15.3)	19 (18.4)	11 (42.3)	
Skin and soft tissue, *n* (%)	17 (4)	14 (4.7)	2 (1.9)	1 (3.8)	
Mucosal-barrier injury-associated BSI, *n* (%)	32 (7.5)	24 (8)	5 (4.9)	3 (11.5)	
Not defined, *n* (%)	75 (17.5)	51 (17)	23 (22.3)	1 (3.8)	
Others, *n* (%)	13 (3)	10 (3.3)	2 (1.9)	1 (3.8)	
High-risk site infection, *n* (%)^b^	199 (46.4)	142 (47.3)	39 (37.9)	18 (69.2)	0.01
Septic shock, *n* (%)	70 (16.3)	50 (16.7)	13 (12.6)	7 (26.9)	0.20
Mechanical ventilation, *n* (%)	65 (15.2)	46 (15.3)	13 (12.6)	6 (23.1)	0.41
Polymicrobial BSI, *n* (%)^c^	86 (20)	63 (21)	20 (19.4)	3 (11.5)	0.50
Variables associated with antimicrobial therapy					
Adequate empirical therapy, *n* (%)	299 (69.7)^d^	238 (79.3)	58 (56.3)	3 (11.5)	<0.001
Combination empirical therapy, *n* (%)	22 (5.1)	18 (6.0)	3 (2.9)	1 (3.8)	0.45
Adequate therapy at any time, *n* (%)	341 (79.5)	247 (82.3)	77 (74.8)	17 (65.4)	0.05
Combination therapy at any time, *n* (%)	19 (4.4)	8 (2.7)	7 (6.8)	4 (15.4)	0.004

Baseline CLCr: baseline creatinine clearance.

^a^Time to BSI: length of hospital stay before BSI onset.

^b^High-risk infection sites included pulmonary, abdominal and catheter-associated infections.

^c^Polymicrobial infection was considered if a microorganism other than an Ecc isolate was also identified in the same blood cultures set that Ecc was recovered (for coagulase-negative *Staphylococcus* spp., BSI was considered only with two positive blood culture sets). A list of copathogens in the three groups can be found in Table S1.

^d^A total of 276 (92.3%) of 299 patients received adequate empirical therapy in the first 24 hours.

Of the 26 CR Ecc isolates, 10 (38.4%) had the detection of a class A carbapenemase (five identified as a KPC), eight (30.7%) had a class B carbapenemase (five identified as NDM) and one (3.8%) had both KPC plus NDM identified. Seven (26.9%) isolates were negative for the presence of carbapenemase (six were recovered from patients before 2018, and one after). A description of year and hospital of recovery of isolates from the three groups are found in Figure S1 (available as Supplementary data at *JAC-AMR* Online).

### 30-day Mortality

The overall 30-day mortality was 22.6% (97 of 429 patients): 20.6% (62/300), 25.2% (26/103) and 34.6% (9/26) in 3GCS, 3GCR and CR Ecc BSIs groups, respectively (*P* = 0.20). The median time to death was 15 days (IQR, 8–27). Nineteen patients (4.4%) died within the first 24 hours. The 30-day mortality according to primary site infection were 29.7% (27 of 91) for abdominal, 35.5% (27 of 76) for respiratory, 21.9% for mucosal-barrier injury-associated BSI (7 of 32), 15.2% for urinary tract (5 of 33) and 18.5% for catheter-associated (17 of 92), and 5.9% (1 of 17) for skin and soft tissue infections. On the basis of these data, we defined high-risk group for mortality those sites with mortality proportions >20%, therefore, encompassing pulmonary, abdominal and mucosal-barrier injury-associated BSI; while low-risk were those with mortality proportions <20%, including urinary tract, catheter-associated BSI and skin and soft tissue infections.

The univariate analysis of the variables associated with 30-day mortality are shown in Table 2. Patients who died were significantly older, had higher Charlson comorbidity index and Pitt bacteraemia scores, and were more frequently at ICU at the onset of BSI compared with survivors. Baseline creatinine clearance was significantly lower among non-survivors. There were also non-significant differences in mortality and hospital of admission. Mortality differed according to infection source, with higher rates observed in abdominal, pulmonary and mucosal-barrier injury-associated BSI sources of infection, further grouped as high-risk sites. Patients with septic shock and requiring mechanical ventilation at the onset of BSI presented a significantly higher 30-day mortality rate. Polymicrobial infections were also significantly more common among non-survivors. The frequency of adequate empirical therapy and adequate therapy at any time was significantly lower in the non-survivors group, whereas the proportion of patients receiving adequate combination therapy at any time was significantly higher. No other variable fulfilled the criteria for inclusion in the multivariate model.

**Table 2.** Univariate analysis of factors and 30-day mortality in patients with Ecc BSIs

Variable	30-day mortality		*P*
	Yes (*n* = 97)	No (*n* = 332)	
Variables associated with patients’ baseline status			
Age, years, mean ± SD	68.7 ± 14.7	58.4 ± 18.2	< 0.001
Sex			0.91
Male, *n* (%)	50 (51.5)	167 (50.3)	
Hospital of admission			0.10
Hospital A, *n* (%)	0 (0)	19 (5.7)	
Hospital B, *n* (%)	46 (47.4)	151 (45.5)	
Hospital C, *n* (%)	27 (27.8)	78 (23.5)	
Hospital D, *n* (%)	24 (24.7)	84 (25.3)	
Charlson comorbidity index, median (IQR)	3 (2–6)	2 (1–3)	< 0.001
Baseline CLCr (mL/min), median (IQR)	42 (25.7–72.5)	85 (42.7–104)	< 0.001
Variables associated with BSI			
Pitt bacteraemia score, median (IQR)	3 (0–8)	0 (0–1)	< 0.001
Time to BSI (days), median (IQR)^a^	15 (7–26)	14 (8–24)	0.74
Resistance profile groups			0.20
3GCS, *n* (%)	62 (63.9)	238 (71.7)	
3GCR, *n* (%)	26 (26.8)	77 (23.2)	
CR, *n* (%)	9 (9.3)	17 (5.1)	
Primary site infection			0.01
Abdominal, *n* (%)	27 (27.8)	64 (19.3)	
Urinary, *n* (%)	5 (5.2)	28 (8.4)	
Catheter-associated, *n* (%)	17 (17.5)	75 (22.6)	
Pulmonary, *n* (%)	27 (27.8)	49 (14.8)	
Skin and soft tissue, *n* (%)	1 (1.0)	16 (4.8)	
Mucosal-barrier injury-associated BSI, *n* (%)	7 (7.2)	25 (7.5)	
Not defined, *n* (%)	12 (12.4)	63 (19)	
Others, *n* (%)	1 (1.0)	12 (3.6)	
High-risk infection site, *n* (%)^b^	61 (62.9)	138 (41.6)	< 0.001
ICU admission, *n* (%)	51 (52.6)	49 (14.8)	< 0.001
Septic shock, *n* (%)	47 (48.5)	23 (6.9)	< 0.001
Mechanical ventilation, *n* (%)	41 (42.3)	24 (7.2)	< 0.001
Polymicrobial infection, *n* (%)^c^	29 (29.9)	57 (17.2)	0.006
Variables associated with antimicrobial therapy			
Adequate empirical therapy, *n* (%)^d^	56 (57.7)	243 (73.2)	0.004
Combination empirical therapy, *n* (%)	6 (6.2)	16 (4.8)	0.60
Adequate therapy at any time, *n* (%)	54 (55.7)	287 (86.4)	< 0.001
Combination therapy at any time, *n* (%)	8 (8.2)	11 (3.3)	0.04

Baseline CLCr: baseline creatinine clearance.

^a^Time to BSI: length of hospital stay before BSI onset.

^b^High-risk infection sites included pulmonary, abdominal and catheter-associated infections.

^c^Polymicrobial infection was considered if a microorganism other than an Ecc isolate was also identified in the same blood cultures set that Ecc was recovered (for coagulase-negative *Staphylococcus* spp., BSI was considered only with two positive blood culture sets). A list of copathogens in the three groups can be found in Table S1.

^d^A total of 276 (92.3%) of 299 patients received adequate empirical therapy in the first 24 hours.

### Multivariate analyses

In the unadjusted model, 3GCR and CR were associated with HRs of 1.10 (95% CI, 0.70–1.73; *P* = 0.70) and 1.42 (95% CI, 0.70–2.86; *P* = 0.33), respectively (Table 3).

**Table 3.** Multivariate analysis of factors associated with 30-day mortality in patients with Ecc BSIs

Variables	*β* coefficient (% change compared with the previous step)	aHR	95% CI	*P*
Unadjusted analysis				
3GCS	reference	reference		
3GCR	0.090 (NA)	1.10	0.70–1.73	0.70
CR	0.349 (NA)	1.42	0.70–2.86	0.33
Step 1				
3GCS		reference		
3GCR	0.132 (46.7%)	1.14	0.72–1.81	0.58
CR	−0.199 (−157.0%)	0.82	0.39–1.7	0.59
Age		1.02	1.01–1.03	0.02
Charlson		1.14	1.05–1.25	0.003
ICU admission		2.89	1.88–4.39	< 0.001
Glomerular filtration rate (mL/min)		0.99	0.98–0.99	0.007
Step 2				
3GCS		reference		
3GCR	0.154 (16.7%)	1.16	0.74–1.85	0.51
CR	−0.147 (−26.1%)	0.86	0.42–1.77	0.69
Charlson Comorbidity Index		1.09	1.01–1.18	0.04
Glomerular filtration rate (mL/min)		0.99	0.98–0.99	< 0.001
Pitt Bacteraemia Score		1.52	1.36–1.69	< 0.001
Polymicrobial infection		2.03	1.3–3.2	0.002
Step 3 (final model)				
3GCS		reference		
3GCR	−0.283 (−283.8%)	0.75	0.46–1.23	0.26
CR	−0.463 (−215.0%)	0.63	0.29–1.32	0.22
Charlson^a^		1.13	1.04–1.22	0.003
Glomerular filtration rate (mL/min)^b^		0.99	0.98–0.99	< 0.001
Pitt Bacteraemia Score^a^		1.23	1.17–1.30	< 0.001
Polymicrobial infection^c^		1.74	1.11–2.72	0.02
Adequate therapy at any time^d^		0.16	0.10–0.27	< 0.001

Data are presented as aHR with 95% CI and corresponding *P*. Stepwise models were adjusted for baseline patient characteristics, infection characteristics, and treatment factors.

Baseline CLCr, baseline creatinine clearance.

^a^Effect estimate for each point in the score.

^b^Effect estimate for each unit in glomerular filtration rate. Categorizing this variable, the aHR of glomerular filtration rate <30 mL/min was 1.77 (95% CI 1.14–2.77), *P* = 0.012.

^c^Polymicrobial infection was considered if a microorganism other than an Ecc isolate was also identified in the same blood cultures set that Ecc was recovered (for coagulase-negative *Staphylococcus* spp., BSI was considered only with two positive blood culture sets). A list of copathogens in the three groups can be found in Table S1.

^d^Adequate empirical therapy was highly associated with adequate therapy at any time: 279 (81.8%) of 341 of patients who received adequate therapy at any time received adequate empirical therapy. Changing adequate therapy at any time by adequate empirical therapy also produced an independent, although less pronounced, protective effect of antimicrobial therapy: aHR, 0.46; 95%CI, 0.29–0.73; *P* = 0.001.

For 3GCR group, adjustment for patients baseline status in step 1 increased the effect estimate [adjusted (aHR), 1.14; 95% CI, 0.72–1.81; *P* = 0.58; 46.7% increase in *β* coefficient compared with the unadjusted value] and remained similar after inclusion of BSI-related covariates in step 2 (aHR, 1.16; 95% CI, 0.74–1.85; *P* = 0.51; 16.7% increase in *β* coefficient compared with step 1). After adjustment for treatment-related variables in final model (step 3), the direction of the effect was inverted (aHR, 0.75; 95% CI, 0.46–1.23; *P* = 0.26), with a high negative change in the *β* coefficient (283.8%) compared with step 2.

For the CR group, adjustment in step 1 inverted the direction of the association (aHR, 0.82; 95% CI, 0.39–1.70; *P* = 0.59; 157% decrease in *β* coefficient compared with the unadjusted value), which remained stable in step 2 (aHR, 0.86; 95% CI, 0.42–1.77; *P* = 0.69; 26.1% decrease in *β* coefficient compared with the unadjusted value). Adjustment in final model (step 3) further increased the magnitude of the protective effect of adequate therapy as evidenced by the low aHR (aHR, 0.63; 95% CI, 0.29–1.32; *P* = 0.22) and a 215.0% negative change in *β* coefficient.

### Sensibility analysis

Since the definition of adequate therapy requires administration of an *in vitro* active antimicrobial agent for at least 2 days, patients must survive long enough to fulfil this criterion, potentially resulting in an immortal-time bias, which tends to overestimate the effect of antimicrobial therapy. Therefore, we performed a sensibility analysis considering all patients who presented this condition as receiving adequate therapy. In our cohort, 19 of 429 patients (4.4%) died within the first 24 hours after BSI onset and therefore could not meet the definition of adequate therapy regardless of the antimicrobial regimen received: 13 in 3GCS, five in 3GCR and one in the CR group. Considering these 19 patients as receiving adequate therapy would not substantially change the effect of adequate antimicrobial therapy at any time in the final model (step 3: aHR, 3.30; 95%CI, 1.96–5.54; *P* < 0.001).

## Discussion

In this large multicentre cohort of patients with healthcare-associated Ecc BSI, we found an overall crude high 30-day mortality (22.6%), which, although not statistically significant, proportionally increased according to the susceptibility profile (20.6% in 3GCS, 25.2% in 3GCR and 34.6% in CR Ecc BSIs). Moreover, we used a hierarchical framework for multivariable model construction to assess the contribution of different groups of variables to the observed associations between resistance profiles and mortality by examining changes in the *β* coefficient across sequential adjustment steps.^37–39^

The overall 30-day mortality observed in our study aligns with previous cohorts of patients with carbapenem-susceptible *Enterobacter* spp. BSIs, which ranged from 14.0% to 25.7% across heterogeneous populations.^40–51^ Fewer studies addressed only Ecc BSI.^39,40,44,47–49,51^ Of these Ecc studies, none evaluated mortality according to the susceptible profile. Notably, only one study reported 30-day mortality (three of four patients) in metallo-*β*-lactamase-producing CR Ecc BSI.^52^ We found an absolute difference in mortality rates of 3GCR compared to 3GCS was relatively small (4.6%), particularly if compared with the difference between CR and 3GCS (14.0%). The lack of statistical significance in this latter comparison was probably due to the lower number of patients with CR Ecc BSI.

In the hierarchized model, when adjusted for baseline characteristics (step 1), the effect remained similar for 3GCR, but with an increase in 46% in the *β* coefficient in comparison with that of unadjusted analysis. This suggests these variables were, in fact, generally worse in 3GCS than in 3GCR ECC BSIs patients. On the other hand, the direction for CR Ecc BSIs was inverted in step 1, and an important modification in the *β* coefficient of CR Ecc was observed (negative change of 147%), indicating that adjustment for these variables substantially attenuated the association between carbapenem resistance and mortality. In other words, CR Ecc BSIs occurred in patients with more unfavourable baseline conditions (as evidenced in Table 1) and may partially account for the higher absolute mortality rates.

When variables associated with presentation of severity of BSIs (step 2) were adjusted for baseline characteristics (step 1), the effect remained similar for both 3GCR and CR Ecc BSIs, although the effect of variables associated with BSI episode (Pitt score and polymicrobial infection) strengthened the association observed for 3GCR isolates and attenuated the effect of CR on mortality. Of note, the Pitt score was highly collinear with high-risk infection sites (data not shown), and only the former was maintained in the model.

Finally, it was observed that certain variables related to antimicrobial therapy substantially modified the estimated effect of third-generation cephalosporin and carbapenem resistance on outcomes in Ecc BSIs, leading to a marked attenuation of the regression coefficients in the multivariable model (both with a negative change in *β* coefficient of >200%). The difference in these variables is clear in Table 1: whereas almost 80% of patients with 3GCS BSIs received adequate empirical therapy, the proportion dropped to 56% in those with 3GCR BSI and to only 11% in patients with CR Ecc BSIs. The proportions of adequate therapy at any time slightly increased in the 3GCS group (82%), while it was more pronounced in 3GCR and CR groups (almost 75% and 65% of adequate therapy at any time, respectively). Indeed, adequate therapy at any time was the variable most strongly associated with a lower risk of mortality (aHR 0.16).

Other variables independently associated with mortality in the final model (step 3) were Charlson and Pitt scores, baseline glomerular filtration rate and polymicrobial BSI. Variables associated with baseline characteristics and severity of BSI presentation, such as septic shock,^40–43^ and Acute Physiology and Chronic Health Evaluation II and Pitt score^40^ have been reported in other studies that addressed risk factors for mortality in Ecc BSIs.

Our study has limitations that should be acknowledged. First, we did not assess the role of specific antimicrobial agents on the outcomes. Therefore, we could only measure a ‘quantitative’ effect of antimicrobial therapy in the final model (step 3), and a potential ‘qualitative’ effect (i.e. use of drugs with lower clinical effectiveness) of therapy was not addressed and cannot be ruled out. It is possible that despite the lower proportions of adequate empirical and at any time therapy in 3GCR and CR groups, drugs with inferior clinical effectiveness (e.g. piperacillin-tazobactam for ESBL-producing 3GCR isolate, aminoglycosides or polymyxins in monotherapy), could be used more often in patients infected by 3GCR isolates, but particularly in CR isolates, hence contributing to the attenuation of the resistance profile effect on mortality in step 3. Another point to highlight in therapy is that combination therapy appears more often in the CR group and is also associated with mortality in univariate analysis. In the absence of novel antimicrobial agents the recommendation for therapy has been the use of two *in vitro* active agents.^53–55^ Very few patients used combination therapy; this strategy was confounded by the indication and it was not our aim to investigate the role of combination, hence the findings of univariate analysis should not be overinterpreted. Third, CLSI criteria (pre-2019) and EUCAST criteria were applied during distinct periods, reflecting the adoption of EUCAST in Brazil in 2018. Notably, had EUCAST been applied in the earlier period (pre-2019), the sole resistance breakpoint modification that would have influenced our findings would have been that for meropenem. Resistance breakpoints of meropenem were lower in CLSI (≥4 mg/L) than in EUCAST (>8 mg/L), which could lead to a misclassification of an isolate as CR instead of 3GCR, if it had been recovered before 2018 and had an MIC = 8 mg/L. However, most isolates produced a carbapenemase, which, although not impossible, makes this misclassification less likely. Of the seven non-carbapenemase-producing isolates, six were recovered from patients before 2018 and one after. Thus, it is theoretically possible that these six isolates would have been assigned to the 3GCR group, if their MIC were 8 mg/L. Nonetheless, four of six patients with these isolates have survived; so, even if all six had an MIC of 8 mg/L, their assignment to the 3GCR group would only accentuate the mortality of the CR group. Fourth, despite being the largest cohort of Ecc BSIs, including in number of CR isolates, there were only 26 patients in this group, and this has impaired the statistical power to detect statistically significant differences in mortality. In addition, changes in effect estimates of this group across hierarchical adjustment steps should be interpreted cautiously, since they may reflect both underlying differences between groups and random variation due to limited statistical power. Fifth, we did not collect data on source control measures, which may be particularly relevant in central line-associated and intra-abdominal BSIs. If such measures were distinct among groups, it might have contributed to the observed outcomes, especially in patients with intra-abdominal infections requiring surgical intervention. Sixth, our definition of adequate therapy required administration of an active antimicrobial agent for at least 2 days. Consequently, patients who died earlier after BSI onset could not fulfil this criterion, introducing the possibility of immortal-time and survivor bias. However, the sensibility analysis ruled out any major modification in the overall results. Finally, we did not perform molecular analysis for the presence of ESBL in 3GCR isolates. Lee *at al*. investigated the impact of ESBL production in 3GCR Ecc BSI and did not find any significant difference.^40^

In conclusion, in this largest multicentre cohort of Ecc BSIs, not statistically significant higher 30-day mortality was observed in patients with BSIs episodes caused by 3GCR and, particularly, CR isolates. Having patients with 3GCS Ecc BSIs as a reference, an analysis of the effect of each group of variables on *β* coefficient of resistance profiles through a hierarchical framework, suggest that higher severity of baseline conditions and less frequent adequate antimicrobial therapy at any time substantially attenuated the association between CR Ecc BSIs and mortality, while adjustment for antimicrobial therapy variables markedly attenuated the association between mortality of patients with 3GCR Ecc BSIs. Improving antimicrobial therapy against 3GCR and CR Ecc isolates may help improve clinical outcomes in patients with Ecc BSIs.

## Supplementary Material

dlag172_Supplementary_Data

## Supplementary data

Figure S1 and Table S1 are available as Supplementary data at *JAC-AMR* Online.
