# Supplementary material for: Impact of third-generation cephalosporin and carbapenem resistance profiles on the mortality of patients with Enterobacter cloacae complex bloodstream infections: a retrospective multicentre cohort study
Source: JAC Antimicrob Resist. 2026 Aug 25;8(4):dlag172. doi: 10.1093/jacamr/dlag172 (PMC13504271; doi:10.1093/jacamr/dlag172)
Supplement: Supplementary file 1 [file dlag172_supplementary_data.pdf]

Supplementary Material

Impact of third-generation cephalosporin and carbapenem resistance profiles on the mortality of patients with *Enterobacter cloacae* complex bloodstream infections: a retrospective multicenter cohort study

Supplementary Table S1. Microorganisms identified in the 86 patients with polymicrobial bloodstream infections.

Copathogen[a]	Total (n=104)	3GCS (n=73, 70.2%)	3GCR (n=28, 26.9%)	CR (n=3, 2.9%)
Klebsiella pneumoniae	18 (17.3)	15 (20.5)	2 (7.1)	1 (33.3)
Escherichia coli	14 (13.5)	11 (15.1)	2 (7.1)	1 (33.3)
Pseudomonas aeruginosa	9 (8.7)	5 (6.8)	3 (10.7)	1 (33.3)
Klebsiella oxytoca	8 (7.7)	8 (11.0)	0	0
Enterococcus faecalis	7 (6.7)	5 (6.8)	2 (7.1)	0
Coagulase-negative staphylococci	7 (6.7)	5 (6.8)	2 (7.1)	0
Staphylococcus aureus	6 (5.8)	5 (6.8)	1 (3.6)	0
Enterococcus spp.	6 (5.8)	4 (5.5)	2 (7.1)	0
Serratia marcescens	5 (4.8)	4 (5.5)	1 (3.6)	0
Acinetobacter baumannii	4 (3.8)	1 (1.4)	3 (10.7)	0

Enterococcus faecium	4 (3.8)	1 (1.4)	2 (7.1)	1 (33.3)
Streptococcus viridans group	2 (1.9)	1 (1.4)	1 (3.6)	0
Klebsiella aerogenes	2 (1.9)	2 (2.7)	0	0
Bacteroides spp.	1 (1.0)	1 (1.4)	0	0
Citrobacter freundii	1 (1.0)	1 (1.4)	0	0
Acinetobacter spp.	1 (1.0)	1 (1.4)	0	0
Proteus mirabilis	1 (1.0)	1 (1.4)	0	0
Enterococcus durans	1 (1.0)	0	1 (3.6)	0
Streptococcus pneumoniae	1 (1.0)	1 (1.4)	0	0
Citrobacter youngae	1 (1.0)	1 (1.4)	0	0
Pseudomonas putida	1 (1.0)	1 (1.4)	0	0
Morganella morganii	1 (1.0)	1 (1.4)	0	0
Acinetobacter sp.	1 (1.0)	0	1 (3.6)	0
Aeromonas spp.	1 (1.0)	1 (1.4)	0	0
Citrobacter koseri	1 (1.0)	0	1 (3.6)	0

Data are presented as n (%). Percentages were calculated using the total number of isolates of each species as the denominator.
[a] Categories are not mutually exclusive because some episodes included more than one copathogen.

Supplementary Figure S1. Temporal and center-specific distribution of third-generation cephalosporin-resistant and carbapenem-resistant *Enterobacter cloacae* complex isolates.

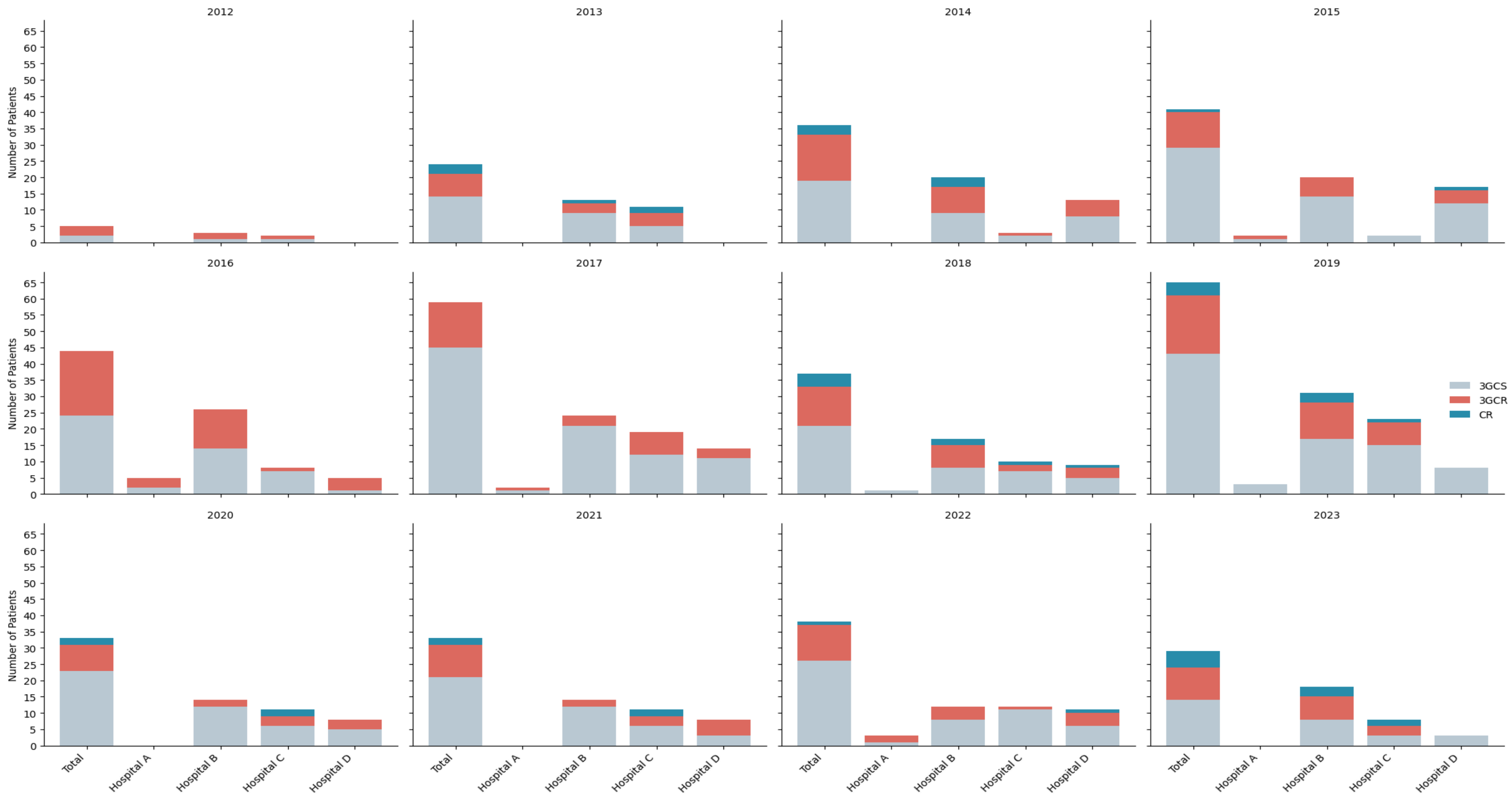

3GCR, third-generation cephalosporin-resistant; CR, carbapenem-resistant; Ecc, *Enterobacter cloacae* complex. Bars indicate the number of bloodstream isolates identified annually in each participating center. Colors represent susceptibility profiles.
